# Gpr177 Deficiency Impairs Mammary Development and Prohibits Wnt-Induced Tumorigenesis

**DOI:** 10.1371/journal.pone.0056644

**Published:** 2013-02-15

**Authors:** Eri Ohfuchi Maruyama, H-M. Ivy Yu, Ming Jiang, Jiang Fu, Wei Hsu

**Affiliations:** Department of Biomedical Genetics, Center for Oral Biology, James P. Wilmot Cancer Center, University of Rochester Medical Center, Rochester, New York, United States of America; Baylor College of Medicine, United States of America

## Abstract

Aberrant regulation of the Wnt pathway, essential for various developmental processes, is tightly linked to human breast cancers. By hijacking this evolutionary conserved signaling pathway, cancer cells acquire sustaining proliferation ability, leading to modification of physiologic properties necessary for tumor initiation and progression. An enormous wealth of knowledge on the importance of Wnt signaling in breast development and cancer has been obtained, but the cell types responsible for production of this proliferative signal operating within normal and malignant tissues remains poorly understood. Here we report that Wnt production mediated by Gpr177 is essential for mammary morphogenesis. The loss of Gpr177 interferes with mammary stem cells, leading to deficiencies in cell proliferation and differentiation. Genetic analysis further demonstrates an indispensable role of Gpr177 in Wnt-induced tumorigenesis. The Gpr177-deficiency mice are resistant to malignant transformation. This study not only demonstrates the necessity of Wnt in mammary organogenesis but also provides a proof-of-principle for targeting of Gpr177 as a potential new treatment for human diseases with aberrant Wnt stimulation.

## Introduction

The evolutionary conserved signal transduction pathways are essential for development of organisms. These signals are conveyed through cell-surface receptors to trigger a cascade of signaling events, which are utilized in diverse developmental processes [1], [2], [3], [4]. Aberrant regulation of these pathways is tightly linked to a variety of congenital diseases and cancers. Wnt is one of these signals necessary for organ development and disease [5], [6]. The ability of Wnt to modulate stem cells has been suggested to be critically involved in mammary development and tumorigenesis [7]. Because of the dynamic changes in morphology, the Wnt-mediated stem cell regulation should be essential for different phases of mammary morphogenesis. Mouse genetic analysis has so far indicated the requirement of Wnt signaling regulators only for prenatal and pregnancy-associated mammary development [8]. Due to overlapping expression of many *Wnts* in the developing mammary gland, gene specific inactivation has not been practical, and has encountered issues related to functional redundancy. There is a lack of evidence to support the necessity of Wnt in puberty-induced development of the mammary gland which involves the dramatic morphological alteration.

The hallmarks of cancer comprise a number of biological capabilities acquired during malignant transformation. Among them, the ability to sustain chronic proliferation is arguably the most fundamental trait of cancer cells [9]. The signals promoting cell growth and division are carefully controlled to ensure a homeostasis of cell number, thereby maintaining normal tissue architecture and function. By hijacking the evolutionary conserved signaling pathways, cancer cells control their own destinies, leading to modulation of biological properties critical for creation of an untamable growing environment [10], [11], [12], [13]. However, the precise identities and sources of these developmental signals, as well as the mechanisms controlling their release, remain largely elusive. Wnt is frequently hijacked by cancer cells to acquire the capability to sustain proliferative signaling [14], [15], [16]. In the mammary gland, Wnt has been proven to be a potent oncogenic inducer [17]. While an enormous wealth of knowledge on the importance of Wnt signaling in development and disease has been obtained, the mechanism underlying Wnt maturation, sorting and secretion has just begun to unfold [18], [19], [20], [21]. Nonetheless, the production of Wnt operating within normal and malignant tissues remains poorly understood.

We have recently identified *Gpr177* as the mouse orthologue of *Drosophila Wls/Evi/Srt*, and showed that this gene product is essential for proper sorting and secretion of Wnt [22], [23], [24]. The abrogation of Wnt production caused by Gpr177 deletion provides an excellent strategy to potentially eliminate all Wnt proteins, and determine the source of Wnt during organogenesis. To further decipher Wnt signaling regulation in mammary development and tumorigenesis, we have created mouse models deficient for Gpr177. The loss of Gpr177 interferes with mammary stem cells, leading to deficiencies in cell proliferation and differentiation. The Gpr177-mediated Wnt production and signaling is essential for mammary morphogenesis. Genetic analysis further indicates an indispensable role of Gpr177 in Wnt-induced tumorigenesis. This study thus demonstrates the requirement of Wnt in mammary maturation, and provides a proof-of-principle for targeting of Gpr177 in human diseases caused by aberrant Wnt stimulation.

## Materials and Methods

### Mouse strains

The Gpr177Fx, MMTV-Cre [Tg(MMTV-Cre)4Mam/J line D], MMTV-Wnt1 [B6SJL-Tg(Wnt1)1Hev/J] and R26RlacZ [B6.129S4-Gt(ROSA)26Sor^tm1Sor^/J] mouse strains and genotyping methods were reported previously [22], [23], [25], [26], [27], [28]. For generating the Gpr177^MMTV^ mouse strain, the MMTV-Cre transgene was bred into the Gpr177Fx homozygous background. The Cre expression led to deletion of exon 3, resulting in a null mutation [23], [29], [30]. To create the MMTV-Wnt1; Gpr177^MMTV^ mutant strain, the MMTV-Wnt1; MMTV-Cre; Gpr177Fx/+ males were crossed with females homozygous for the Gpr177Fx allele. All mice were kept and analyzed as nulliparous females. This study was carried out in strict accordance with the recommendations in the Guide for the Care and Use of Laboratory Animals of the National Institutes of Health. Care and use of experimental animals described in this work is approved by the University Committee on Animal Resources at the University of Rochester (Protocol Number 2002–213).

### Cells

The procedures for isolation of primary mammary epithelial cells were described previously [22]. For mammosphere culture, a single cell suspension of mammary epithelial cells was cultured in DMEM/F12 media containing 2% B27, 20 ng/ml of EGF, 20 ng/ml of bFGF, 5 µg/ml of insulin, 500 ng/ml of hydrocortisone, and 4 µg/ml of heparin. For inhibition of Wnt production, 5 µM IWP-2 (Santa Cruz, Santa Cruz, CA) was present in the culture media. The formation of spheres was analyzed after 7 days. Mammospheres were treated with 0.25% Trypsin for 5 min at 37°C, and physically dissociated into single cell suspension for culturing the subsequent passage. For Wnt secretion analysis, mouse L were cultured in DMEM media containing 10% fetal bovine serum. The stably transformed cell line, L-Wnt3a, was cultured with the addition of 0.4 mg/ml G418. Cells were cultured without G418 for 4 days to collect the condition media, which were harvested and filtered through a nitrocellulose membrane. To knockdown Gpr177, cells were transfected with 50 nM Gpr177 siRNA (Thermo Scientific, Fremont, CA, USA) using Lipofectamine RNAiMAX (Invitrogen, Carlsbad, CA, USA). Cell culture media were then changed after 24 hours to collect the condition media. For TOPFLASH reporter analysis, the collected condition media were used to culture the signal-receiving cells transfected with the TOPFLASH reporter (Upstate Biotechnology, Boston, MA, USA). Plasmid DNA transfection and luciferase analysis were performed as described [22], [31].

### Mammary staining, β-gal staining, histology, immunostaining and immunoblot

The number 4 mammary glands were dissected at the indicated times of development for whole mount staining as described [32]. Briefly, the glands were hydrated and stained in carmine alum solution overnight after fixation in Carnoy's fixative for at least 4 hours at room temperature. Samples were then dehydrated and cleared in Histoclear. Details for β-gal staining in whole mounts or sections were described previously [33], [34]. For histological evaluation, tissues were dissected, fixed in 10% buffered formalin and paraffin embedded to obtain sections which were stained with hematoxylin/eosin. Sections were subject to immunostaining with avidin∶biotinlylated enzyme complex as described [27], [31], [35], [36], [37]. The immunological staining was visualized by enzymatic color reaction, fluorescence or electron microscopy. Immunoblot analysis was performed as described [37]. Bound primary antibodies were detected with horseradish peroxidase-conjugated secondary antibodies (Vector Laboratories, Burlingame, CA, USA), followed by ECL-mediated visualization (GE HealthCare, Waukesha, WI, USA) and autoradiography. Mouse polyclonal antibody Keratin 6 (Covance, Emeryville, CA, USA); mouse monoclonal antibodies ABC (Millipore, Billerica, MA, USA), Keratin 14 (Thermo Scientific), and SMA (Thermo Scientific); rabbit polyclonal antibodies Gpr177 [22], [24], and phosphorylated Histone H3 (Cell Signaling, Danvers, MA); rabbit monoclonal antibodies Ki67 (Thermo Scientific), cyclin D1 (Thermo Scientific), and Keratin 18 (Thermo Scientific); goat polyclonal antibodies Wnt1 (Thermo Scientific and R&D Systems, Minneapolis, MN, USA) and Wnt3/3a (Santa Cruz) were used as primary antibodies in these analyses. Controls for immunostaining without the primary antibodies are shown in Figure S3. Images were taken using Nikon SMZ1500 or TS100-F microscope (Nikon, Melville, NY, USA) connected to a SOPT RT camera (Diagnostic Instruments, Sterling heights, MI, USA), and Zeiss Axio Observer microscope and Imager (Carl Zeiss, Thornwood, NY).

### Mammary Transplantation

Primary mammary epithelial cells were freshly isolated and collected into sterile screw-cap tubes. Cell pellets were suspended in fresh PBS buffer, counted and re-suspended in PBS buffer at appropriate cell densities. Mammary epithelial cells were then transplanted into the cleared number 4 mammary fat pad of 3 week old SCID mice. Six weeks after transplantation, the recipient fat pad were dissected and analyzed by whole mount mammary gland staining with carmine red. Limiting dilution data were analyzed by ELDA as described [38].

## Results

### Gpr177 is essential for mammary development

**Figure 1:**
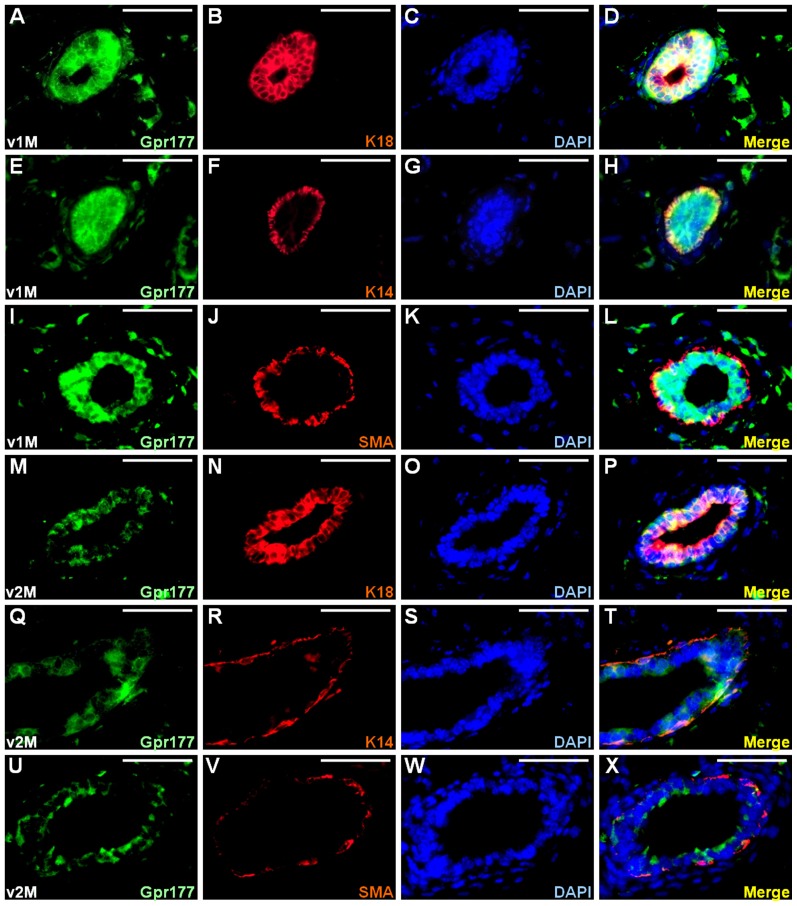
Gpr177 is dynamically expressed in mammary development. The expression of Gpr177 in the luminal and basal/myoepithelial cells was analyzed by co-immunostaining with K18 (A–D, M–P), K14 (E–H, Q–T), and SMA (I–L, U–X), respectively. Sections of the virgin 1 month (v1M; A–L) and 2 month (v2M; M–X) mammary gland were co-immunostained with Gpr177 (green) and cell type-specific marker (red) and counterstained with DAPI (blue). Scale bars, 50 µm (A–X).

We hypothesized that reciprocal regulation of Gpr177 and Wnt is required for the formation of various organs, including the mammary gland. To test our hypothesis, we first examined the expression of Gpr177. The specificity of the antibody against Gpr177 was demonstrated in our prior studies [22], [23], [24]. In the developing mammary gland at one month, co-immunostaining revealed that Gpr177 was expressed in the luminal epithelial cells positive for K18 (Figure 1A–D). The expression of Gpr177 was also co-localized with K14-expressing basal/myoepithelial cells (Figure 1E–H). However, this co-localization pattern was not identical to that of SMA, which is another basal/myoepithelial marker (Figure 1I–L). In the mature mammary gland which has finished branching morphogenesis at two months, the number of epithelial cells expressing Gpr177 and its expression level were significantly reduced (Figure 1M, Q, U) compared to that at one month (Figure 1A, E, I). At this stage, the co-localization of Gpr177 with K18 (Figure 1M–P), but not K14 and SMA (Figure 1Q–X), remained detectable. These data suggest a potential involvement of Gpr177 in the rapid growth phase of mammary gland during puberty.

Next, we created mouse models with conditional inactivation of *Gpr177* to definitively assess its requirement in mammary morphogenesis. The Gpr177Fx allele was crossed with the MMTV-Cre transgene to generate the MMTV-Cre; Gpr177Fx/+ line. Intercross between the MMTV-Cre; Gpr177Fx/+ mice and the Gpr177Fx/Fx mice obtained the MMTV-Cre; Gpr177Fx/Fx (Gpr177^MMTV^) mutants. The Gpr177Fx/+, Gpr177Fx/Fx and MMTV-Cre; Gpr177Fx/+ littermates were used as experimental control. In the Gpr177^MMTV^ mutants, *Gpr177* was inactivated by the MMTV-Cre transgene through Cre-mediated recombination. Using the R26R reporter allele, lineage tracing analysis revealed the efficiency of Cre-mediated recombination in the developing mammary gland at postnatal stages (Figure S1A–I). Double labeling of the β-gal positive cells with K18, K14 or SMA further showed that Cre recombination occurs in all mammary cell types (Figure S1J–O). Although it has been widely accepted that K14 and SMA are expressed in the same population, we found that certain SMA positive cells do not express K14. This was evident at the terminal end bud (TEB) which is a specialized and highly proliferative structure, containing mammary stem cells (MaSCs) required for ductal elongation during sexual maturation [39]. While the K14 and SMA expression patterns look similar at the mammary duct (Figure S1K, L), an outer layer of cells positive for SMA are clearly negative for K14 at the TEB (Figure S1N, O).

**Figure 2:**
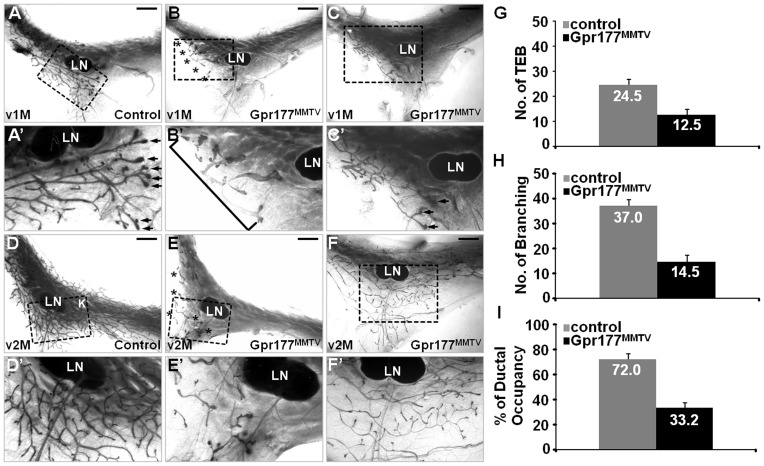
Gpr177 is essential for mammary morphogenesis. Whole mount staining of the number four mammary glands reveals severe developmental defects associated with inactivation of Gpr177 by MMTV-Cre (Gpr177^MMTV^) at virgin 1 month (v1M; A–C) and virgin 2 month (v2M; D–F). Asterisks and bracket indicate the residual epithelial components detected in the Gpr177^MMTV^ mutant. Arrows indicate the presence of TEB in the v1M control littermates. Enlargements of the insets (A–F) are shown in A′–F′. LN, lymph node. The average number of TEB (G) and branching (H), as well as the percentage of ductal occupancy (I), were examined in control (genotype: MMTV-Cre; Gpr177Fx/+, Gpr177Fx/Fx or Gpr177Fx/+) and Gpr177^MMTV^ for quantitative analysis (n = 3). Scale bars, 2 mm (A–F).

The Gpr177^MMTV^ mutants exhibited severe defects in mammary development (Figure 2 and S2). At birth, the mammary gland normally ramifies into a small mammary tree with 15–20 branching ducts, remaining static until puberty. No obvious difference was found in the newborn Gpr177^MMTV^ gland (Figure S2A, B; 100%, n = 3). However, reduced ductal branches were often shown in the mutants before the onset of puberty-dependent development at P14 (Figure S2C, D; 88%, n = 17) and P21 (Figure S2E, F; 50% n = 10). At one month, no TEB and extensive ductal elongation and branching across the lymph node region were detected in 55% of the mutants (Figure 2A, A′ B, B′; 55%, n = 11). In the remaining 45%, the number of TEB was significantly reduced with branching deficiency (Figure 2C, C′; 45%, n = 11). The difference in phenotypic severity is likely due to effectiveness of MMTV-Cre. The loss of Gpr177 also impaired development of the mature mammary ductal tree, which spreads throughout the fatty stroma at two months (Figure 2D, D′). There were very few epithelial components left in the more severe mutants (Figure 2E, E′; 38%, n = 8). In the less affected ones, the residual TEB was capable of mediating ductal elongation although the branching process was obviously impaired (Figure 2F, F′; 62%, n = 8). Statistically analysis further indicated that the average numbers of TEB and branching were reduced from 24.5% and 37.0% in the control to 12.5% and 14.5% in the Gpr177^MMTV^ mutant, respectively (Figure 2G, H). The loss of Gpr177 also caused a reduction on the percentage of ductal occupancy from 72.0% in the control to 33.2% in the mutant (Figure 2I).

### Disruption of Gpr177 impairs cell proliferation and differentiation

**Figure 3:**
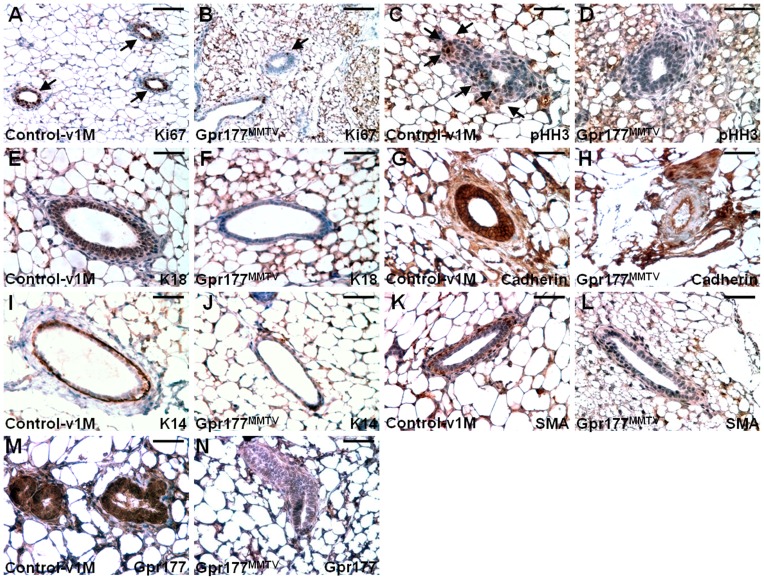
The loss of Gpr177 impairs mammary cell proliferation and differentiation. Cells undergoing mitotic division are detected by immunostaining of Ki67 (A, B) and pHH3 (C, D) in the mammary gland at one month. Sections were immunostained with the antibody (brown) and counterstained with Hematoxylin (blue). Arrows indicate cells positive for immunostaining. Immunostaining of K18 (E, F), Cadherin (G, H), K14 (I, J), SMA (K, L) and Gpr177 (M, N) characterizes the effect of the Gpr177 deletion on specification of mammary cell types. Scale bars, 100 µm (A, B); 50 µm (C–N).

To elucidate the mechanisms underlying the mammary defects of Gpr177^MMTV^, we investigated cell proliferation and differentiation affected by the Gpr177 ablation. During puberty-mediated mammary development, mitotic division was impaired in the mutants. The loss of Gpr177 reduced the number of Ki67 and phosphorylated Histone H3 (pHH3) positive cells at one month (Figure 3A–D). Immunostaining of K14, K18, SMA and cadherin then examined the specification of mammary cell types (Figure 3E–L). The staining of K18 and cadherin indicated an impairment of luminal epithelial differentiation caused by the Gpr177 deletion (Figure 3E–H). Similarly, the differentiation of basal/myoepithelial cells positive for K14 and SMA were significantly affected (Figure 3I–L). Immunostaining of Gpr177 showed the effectiveness of the Cre-mediated deletion (Figure 3M, N). The results suggest that Gpr177 may possess cell autonomous and non-cell autonomous effects on development of luminal and basal/myoepithelial cells.

### The Gpr177-mediated regulation of Wnt is essential for mammary morphogenesis

**Figure 4:**
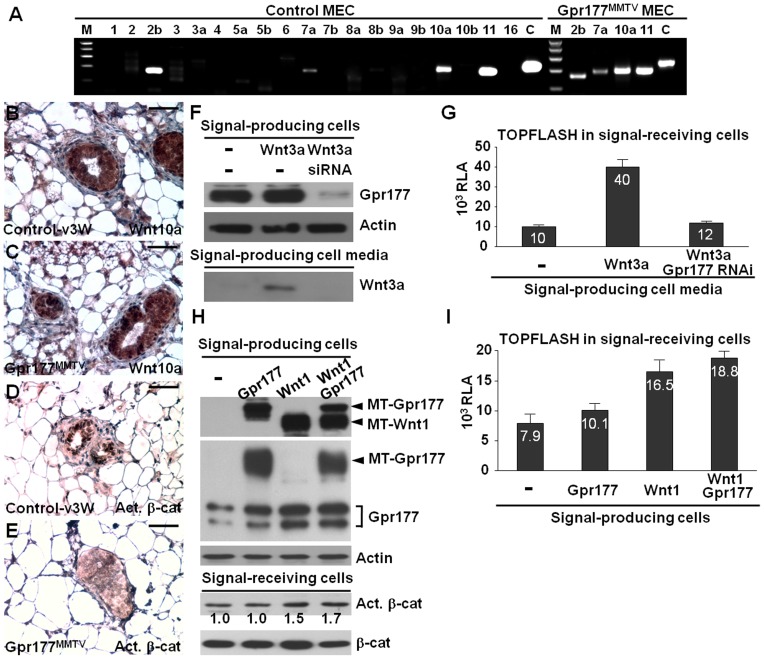
Gpr177 regulates Wnt production and signaling essential for mammary development. (A) RT-PCR analyzes the Wnt transcripts present in the primary mammary epithelial cells (MEC) isolated from the P21 control and Gpr177^MMTV^ glands. Immunostaining of Wnt10a (B, C) and activated β-catenin (Act. β-cat, D, E) reveals Wnt signaling, but not Wnt expression, affected by Gpr177 deficiency. (F–I) Wnt production and signaling are analyzed by the use of mouse L (-) and L-Wnt3a (Wnt3a) cell lines. (F) Immunoblot analysis reveals that the secretion of Wnt is prohibited by the knockdown of Gpr177 in the signal-producing cells. (G) Relative luciferase activity (RLA) analysis of the signal-receiving cells, harboring the TOPFLASH reporter, detects activation of the canonical Wnt pathway in the signal-producing cell media. (H) Immunoblot analysis shows the transient expression of the myc tagged Wnt1 (MT-Wnt1) and myc-tagged Gpr177 (MT-Gpr177), as well as the endogenous Gpr177 (Gpr177) in the signal-producing cells, and the level of Act. β-cat in the signal-receiving cells. The level of actin and total β-catenin (β-cat) is used as loading control. (I) RLA analysis of TOPFLASH examines activation of Wnt/β-catenin signaling in the condition media. Scale bars, 50 µm (B–E).

We further investigated whether Wnt production and signaling are affected by the Gpr177 deletion to elucidate the mechanism underlying mammary development. Genetic inactivation of *Gpr177* had no significant effect on the expression of Wnt mRNAs and proteins in the signal-producing cells, but inhibited canonical Wnt signaling in the signal-receiving cells (Figure 4). The deletion of Gpr177 did not alter the presence of Wnt2b, Wnt7a, Wnt10a and Wnt11 transcripts in the primary mammary epithelial cells (Figure 4A). Similarly, strong expression of Wnt10a protein was also detected in the control and Gpr177^MMTV^ mutant glands (Figure 4B, C). However, nuclear staining of mammary cells with activated β-catenin was greatly abolished in the mutants (Figure 4D, E).

As a Wnt trafficking regulator, Gpr177 is likely involved in Wnt sorting and secretion during mammary morphogenesis. To determine the essential role of Gpr177 in the production of Wnt, we established a Wnt-secretion assay using mouse L and L-Wnt3a cell lines. Wnt3a was detected in the L-Wnt3a conditioned media which were used to culture the signal-receiving cells, mouse L cells containing the TOPFLASH reporter, thereby stimulating its activity (Figure 4F, G). When Gpr177 was knocked down by RNA interference, Wnt3a was not detectable in the L-Wnt3a media (Figure 4F), leading to no stimulation of TOPFLASH in the signal-receiving cells (Figure 4G). Wnt overexpression in the signal-producing cells alone seemed to be sufficient to effectively induce the TOPFLASH reporter in the signal-receiving cells (Figure 4H, I). This is most likely attributed to Gpr177, itself a transcriptional target of Wnt/β-catenin [22]. Because high levels of Wnt are able to promote the expression of endogenous Gpr177 in a positive feedback loop [22], it is not necessary to express Gpr177 exogenously. In cells expressing high levels of Wnt protein, the expression of Gpr177 was elevated (Figure 4H). These results suggest an important role of Gpr177 in Wnt sorting and secretion during mammary morphogenesis.

### Gpr177 is essential for Wnt-dependent regulation of mammary stem cells

**Figure 5:**
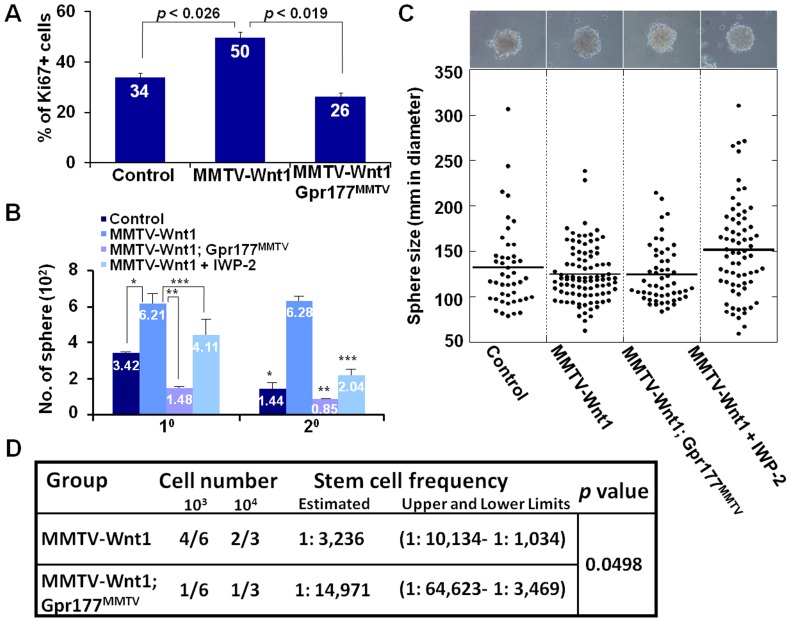
The Wnt-mediated proliferation and self-renewal of mammary stem cells (MaSCs) requires Gpr177. (A) Statistical analysis shows the percentage of mitotic cells positive for immunostaining of Ki67 in the control, MMTV-Wnt1, MMTV-Wnt1; Gpr177^MMTV^ mammary gland. *P* values indicate significance of the study (n = 3). (B) Mammosphere analysis examines the self-renewing and proliferation abilities of MaSCs in primary (1^0^) and secondary (2^0^) cultures of control, MMTV-Wnt1 and MMTV-Wnt1; Gpr177^MMTV^. The effect of IWP-2 on the Wnt-mediated stem cell self-renewal and proliferation is also investigated. Data represent the mean ± SEM in three independent experiments. Asterisks indicate the *p* value (1^0^: *, <0.0062, **, <0.0022, ***, <0.0283 and 2^0^: *, <0.0004, **, <0.0024, ***, <0.0076). (C) Diagram illustrates the average size of spheres is not significantly different in the control (131.9±6.9), MMTV-Wnt1 (124.8±3.2), MMTV-Wnt1; Gpr177^MMTV^ (124.2±4.3), MMTV-Wnt1+IWP-2 (151.5±6.4) cultures. (D) Diagram illustrates the limiting dilution transplantation and calculation of the success ratio showing the frequency of mammary stem cells in MMTV-Wnt1 and MMTV-Wnt1; Gpr177^MMTV^ mice.

The essential role of Gpr177 in mammary morphogenesis prompts us to examine its involvement in cell growth and division associated with Wnt activation. In the MMTV-Wnt1 transgenic mice, we found that the enhanced mitotic divisions caused by aberrant Wnt expression were alleviated in the MMTV-Wnt1; Gpr177^MMTV^ glands (Figure 5A). Gpr177 deficiency had protection against Wnt-induced proliferation, providing a mean to investigate its role in MaSCs where Wnt signaling might be crucial for their developmental regulation. The number of Lin^−^CD29^high^CD24^+^ precursor cells was enhanced in the MMTV-Wnt1 transgenic mice [40]. This population enriched with MaSCs might contribute to the neoplastic transformation induced by Wnt. To test if alterations in stem cell properties contribute to aberrant proliferation of mammary cells, we investigated the self-renewal and proliferating abilities of MaSCs modulated by the Wnt-Gpr177 regulatory pathway. Mammary sphere analysis showed that Wnt overexpression drastically increased the number of spheres formed in the primary and secondary cultures (Figures 5B). However, this abnormality is alleviated by the deletion of Gpr177, implying its indispensable role in Wnt-mediated stem cell regulation. To further examine whether this phenotypic alleviation is due to the important function of Gpr177 in Wnt production, a small molecule inhibitor of Porcupine, IWP-2, was used to block lipid modification of Wnt proteins necessary for their maturation [41]. Similar inhibitory results were obtained by the use of IWP-2 in the sphere analysis, suggesting that the Gpr177-mediated production of Wnt is necessary for MaSC self-renewal and proliferation (Figure 5B). Contrary to the number, the size of mammospheres was not significantly affected by alteration of Wnt signaling (Figure 5C). This is most likely due to the size of spheres determined by not only the proliferative ability of the cells, but also other factors, including their self-renewing ability and sensitivity to growth factors [42]. In contrast to the proliferation analysis in mice (Figure 5A), the supply of growth factors is not limited in cultures. To definitively assess the requirement of Gpr177 for MaSC regulation, we performed limiting dilution transplantation with primary mammary epithelial cells isolated from MMTV-Wnt1 and MMTV-Wnt1; Gpr177^MMTV^ mice. The success ratio of transplantation and calculation was examined by extreme limiting dilution analysis (ELDA) software to determine the frequency of MaSCs [43]. The results showed that the stem cell frequency is 1 in 3×10^3^ for MMTV-Wnt1 (Figure 5D), much higher than the wild type (1 in 4×10^4^) reported previously [44]. However, this increase caused by overexpression of Wnt1 was significantly alleviated by the Gpr177 deletion (1 in 1.5×10^4^ for MMTV-Wnt1; Gpr177^MMTV^, *p* value = 0.0498). We were unable to determine the stem cell frequency of Gpr177^MMTV^ due to low numbers of mammary epithelial cells present in these mutants. The finding indicates an essential role of Gpr177 in Wnt-mediated regulation of MaSCs, further suggesting that targeting Gpr177 for the inhibition of Wnt production in the stem cells might be an effective treatment strategy.

### Inhibition of Gpr177-mediated Wnt production prevents mammary hyperplasia

**Figure 6:**
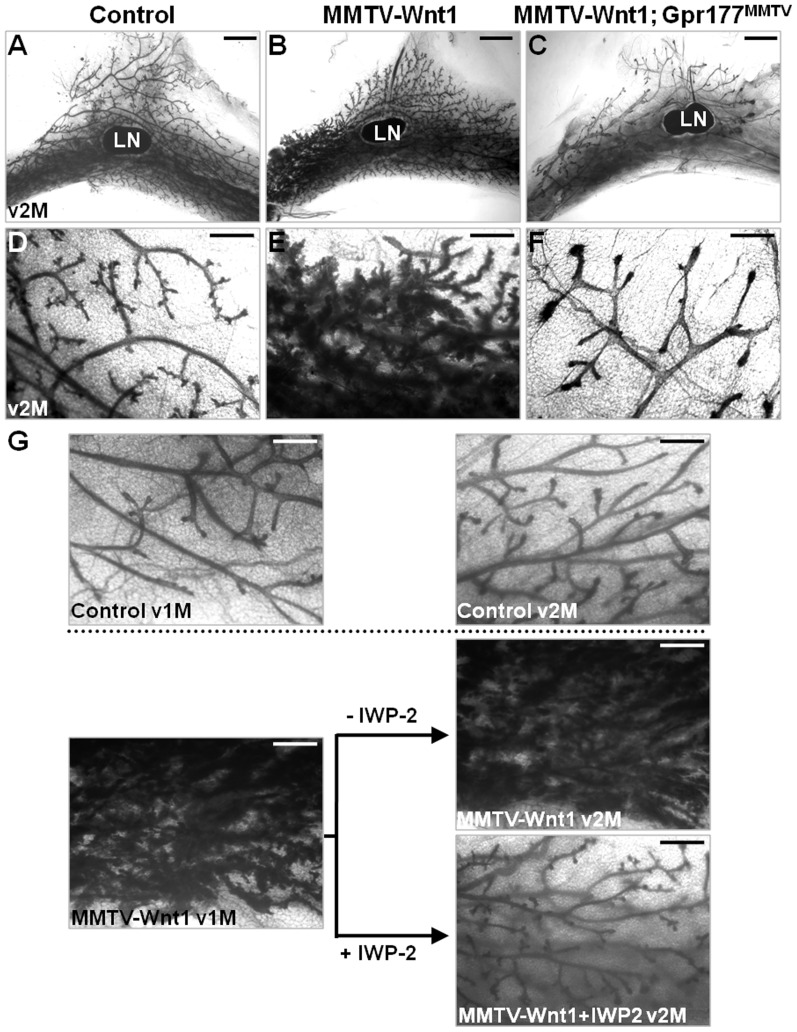
Gpr177-mediated Wnt production is essential for Wnt-induced mammary hyperplasia. (A–F) Whole mammary gland staining of the virgin 2 month (v2M) control (A, D; genotype: Gpr177Fx/+), MMTV-Wnt1 (B, E; genotypes: MMTV-Wnt1; Gpr177Fx/+, MMTV-Wnt1; Gpr177Fx/Fx or MMTV-Wnt1; MMTV-Cre; Gpr177Fx/+), MMTV-Wnt1; Gpr177^MMTV^ (C, F; genotypes: MMTV-Wnt1; MMTV-Cre; Gpr177Fx/Fx) littermates reveals that inactivation of *Gpr177* prohibits mammary hyperplasia caused by Wnt1 overexpression. (G) Whole mount staining of the number four mammary glands shows evidence of mammary hyperplasia in the MMTV-Wnt1, but not control, mice at v1M and v2M. Injection of IWP-2 (250 nmol) once every three days for thirty days is able to prevent the hyperplastic phenotype caused by aberrant expression of Wnt1 (n = 5/6). LN, lymph node. Scale bar, 2 mm (A–C); 500 µm (D–G).

To further our study on the effectiveness of targeting Gpr177 as a potential treatment of diseases caused by Wnt stimulation, we performed in vivo analysis. Aberrant expression of Wnt induces mammary hyperplasia found in all MMTV-Wnt1 mice which provide an excellent model to test this hypothesis [17]. To test if Gpr177 is essential for the premalignant lesions induced by elevated levels of Wnt, a genetic study was carried out by crossing MMTV-Wnt1 into the Gpr177^MMTV^ background. Intercross between the MMTV-Wnt1; MMTV-Cre; Gpr177Fx/+ mice and the Gpr177Fx/Fx mice generated the control (genotype: Gpr177Fx/+); MMTV-Wnt1 (genotypes: MMTV-Wnt1; Gpr177Fx/+, MMTV-Wnt1; Gpr177Fx/Fx or MMTV-Wnt1; MMTV-Cre; Gpr177Fx/+) and MMTV-Wnt1; Gpr177^MMTV^ (genotype: MMTV-Wnt1; MMTV-Cre; Gpr177Fx/Fx) mice. Mammary hyperplasia did not occur in the MMTV-Wnt1; Gpr177^MMTV^ mutants (100%, n = 10), suggesting that the pathogenic effects initiated by Wnt is abolished by genetic inactivation of *Gpr177* (Figure 6A–F). This phenotypic alleviation was not caused by the MMTV-Cre transgene as littermates carrying MMTV-Wnt1 and MMTV-Cre also developed hyperplasia. The mammary phenotype of MMTV-Wnt1; Gpr177^MMTV^ (Figure 6C, F) were reminiscent to some of the Gpr177^MMTV^ mutants (Figure 2F, F′) which further indicates an essential role of Gpr177 in Wnt production necessary for the hyperplastic development of MMTV-Wnt1.

Next, we use IWP-2 to examine whether inhibiting the function of Gpr177 in Wnt production is responsible for this phenotypic alleviation. The hyperplastic transformation were evident in all MMTV-Wnt1 at one month (Figure 6G). We then administrated IWP-2 by peritoneal injection once every three days. The hyperplastic phenotype of MMTV-Wnt1 was prevented after the IWP-2 treatment for one month (Figure 6G). Inhibition of Wnt production was able to correct the hyperplasia, suggesting a treatment potential for diseases caused by Wnt stimulation (83.3%, n = 6). Together with the genetic study, our findings imply that the Gpr177-mediated production of Wnt is necessary for mammary development in health and disease.

### The loss of Gpr177 alleviates the oncogenic effects caused by Wnt stimulation

**Figure 7:**
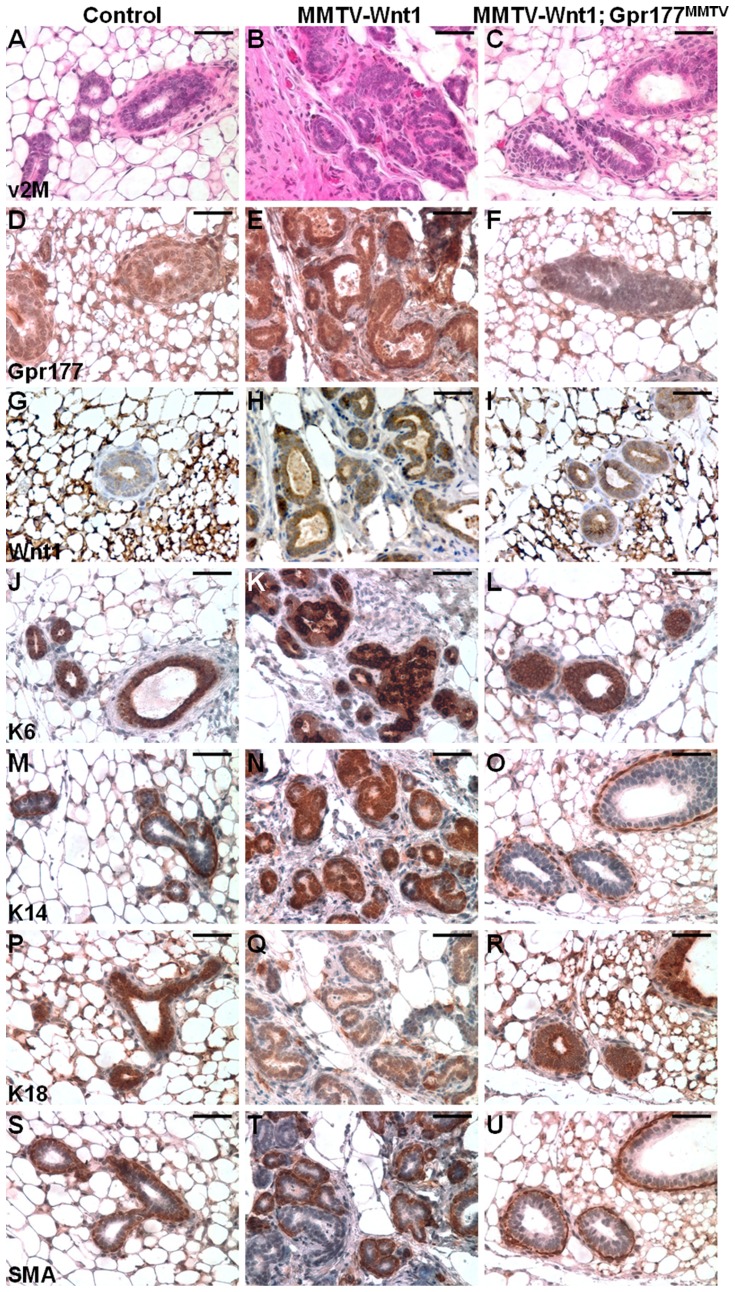
Gpr177 deficiency alleviates the abnormalities of mammary cell types caused by aberrant Wnt expression. (A–U) Sections of the virgin 2 month (v2M) control (A, D, G, J, M, P, S; genotype: Gpr177Fx/+), MMTV-Wnt1 (B, E, H, K, N, Q, T; genotypes: MMTV-Wnt1; Gpr177Fx/+, MMTV-Wnt1; Gpr177Fx/Fx or MMTV-Wnt1; MMTV-Cre; Gpr177Fx/+), and MMTV-Wnt1; Gpr177^MMTV^ (C, F, I, L, O, R, U) are analyzed by H&E staining (A–C) and immunostaining of Gpr177 (D–F), Wnt1 (G–I), K6 (J–L), K14 (M–O), K18 (P–R) and SMA (S–U). Scale bar, 50 µm (A–U).

Molecular analysis then examined if the cellular architecture phenotypes of MMTV-Wnt1 were also alleviated by the Gpr177 deletion (Figure 7A–C). First, we examined the expression of Gpr177 to assess its involvement in the Wnt-induced mammary tumorigenesis. Gpr177 was sparsely expressed at low levels in the mature resting mammary glands at 2 month (Figure 7D). In contrast, a uniform and strong expression of Gpr177 was detected in the mammary hyperplasia, developed in all MMTV-Wnt1 mice (Figure 7E). However, no elevated and uniform expression of Gpr177 was detected in the MMTV-Wnt1; Gpr177^MMTV^ mutants (Figure 7F). Consistent with our previous findings [22], [23], [30], the loss of Gpr177 did not seem to interfere with the expression of the Wnt1 transgene (Figure 7G–I). We next assess the differentiation of mammary cell types. The expression of K6 has been implicated in a subset of mammary progenitor cells [45] which have been implicated in Wnt-induced tumorigenesis (Figure 7J, K). It has also been postulated that MMTV-Wnt1 induces basal cell-like tumors because of the strong expression of K14 (Figure 7M, N). These results are in agreement with the previous observation on MMTV-Wnt1 MaSCs expressing high levels of K6 and K14 [45], [46]. In the MMTV-Wnt1; Gpr177^MMTV^ mutants, we did not detect any aberrant expression of K6 and K14 induced by MMTV-Wnt1 transgene (Figure 7J–O). In contrast, high levels of Wnt1 greatly reduced the luminal epithelial expression of K18 while its expression was comparable in the control and MMTV-Wnt1; Gpr177^MMTV^ mammary glands (Figure 7P–R). Examination on the expression of SMA revealed that only a portion of cells exhibit high levels of expression in the MMTV-Wnt1 mutants (Figure 7T). The lack of SMA expression associated with neoplastic transformation in the MMTV-Wnt1 mice was also alleviated by the Gpr177 ablation (Figure 7S–U).

**Figure 8:**
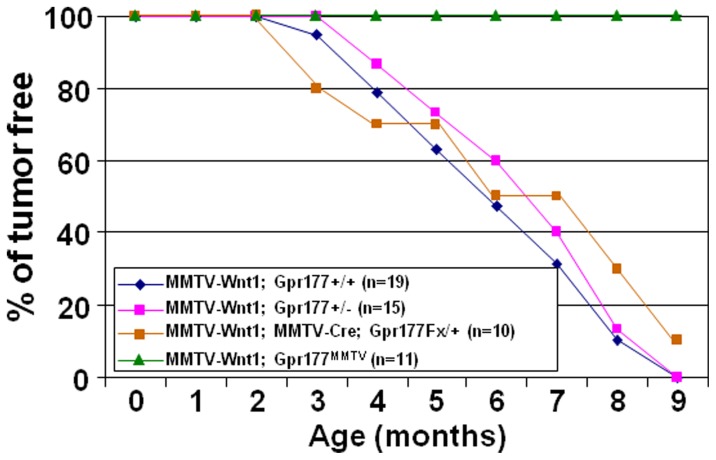
Inactivation of Gpr177 abolishes mammary tumorigenesis induced by Wnt. Graph represents the analysis of tumor development in mice, carrying MMTV-Wnt1 transgene in Gpr177+/+ (n = 19), Gpr177+/− (n = 15), MMTV-Cre; Gpr177Fx/+ (n = 10) and MMTV-Cre; Gpr177Fx/Fx (Gpr177^MMTV^; n = 11) backgrounds in a nine-month observation period.

To definitively examine the role of Gpr177 in Wnt-induced tumorigenesis, we performed tumor development study in a 9-month observation period (Figure 8). Within this period, no mice carrying MMTV-Wnt1 transgene either in the Gpr177+/+ (0%, n = 19), Gpr177+/− (0%, n = 15), or MMTV-Cre; Gpr177Fx/+ (10%, n = 10) background was tumor free with only one exception. A MMTV-Cre; Gpr177Fx/+ female remained tumor free at 9 months but did develop a tumor later. However, we did not detected any tumors in the 9-month old of MMTV-Wnt1; Gpr177^MMTV^ mice (100%, n = 11), implying that Gpr177 deficiency prohibit Wnt-induced tumorigenesis.

## Discussion

This study demonstrates that Gpr177 is essential for Wnt-mediated development of the mammary gland in health and disease. Although many Wnts are expressed in different cell types and phases of mammary development, very little is known about their precise roles in the developmental processes. None of the Wnt knockout mice has shown such severe defects in mammary development similar to the Gpr177 ablation. This is most likely attributed to early lethality associated with the mouse knockout studies as well as functional redundancy caused by the overlapping expression pattern of Wnts. Nonetheless, there is no question that Wnt signaling is intimately involved in breast development and cancer. Using MMTV-Cre to inactivate *Gpr177*, our data suggest that the Gpr177-mediated production of Wnt is essential for mammary morphogenesis. Wnt signal is required for development of the mammary ductal tree at puberty. Wnt likely induces cell autonomous and non-cell autonomous effects on mammary development. Even though Gpr177 has only been shown to regulate Wnt [47], [48], we cannot rule out the possibility that the Gpr177 deletion causes indirect effects on other growth factors. Alternatively, basal/myoepithelial cell differentiation may be affected by the lack/reduced number of the luminal epithelial cells caused by the Gpr177 deletion. Such phenotypes associated with the loss of Gpr177 may indirectly affect the microenvironment necessary for mammary morphogenesis. Furthermore, whether the Gpr177-mediated Wnt regulation plays a role in the stroma to modulate the morphogenetic processes remains to be determined.

Although many *Wnt* genes are expressed during mammary ductal elongation and branching, genetic analysis so far has only linked Wnt5a to these developmental processes. Because mouse knockout of Wnt5a led to perinatal death, the implantation study was used to show that epithelial removal of Wnt5a accelerates mammary development [49]. The stromal cells showed high expression of Wnt5a but its presence there seemed to be dispensable. Our findings suggest that epithelial deletion of Gpr177 does not cause defects resembling, but opposite to, the Wnt5a deletion. It is conceivable that the loss of other Wnt(s) is responsible for the development defects of Gpr177^MMTV^. Alternatively, the observed mammary phenotype represents the sum of all Wnts lost. The role of Gpr177 as a potential master regulator for Wnt sorting and secretion provides an excellent tool to disrupt the production of all Wnt proteins expressed in any given cell. Mice with cell type-specific inactivation of *Gp177* are also excellent models to determine the source of Wnt essential for developmental and pathogenic processes.

Disruption of negative regulators or stimulation of positive regulators, leading to aberrant activation of the canonical Wnt pathway, has been repeatedly implicated in human breast cancers, especially in basal-like carcinomas, one of the most aggressive forms of the disease [6], [50]. The MaSC population is highly increased in the hyperplastic mammary gland of MMTV-Wnt1 [40]. A cancer stem cell population similar to that defined in human breast cancers is also present in the subsequent carcinomas [51]. MMTV-Wnt1 transgenic mice thus provide a powerful tool for the study of Wnt signaling and stem cell characteristics in mammary tumorigenesis [17], [45], [52]. While high levels of canonical Wnt3a have been shown to promote the self-renewal or expansion of MaSCs [53], their contribution to the subsequent malignant transformation remains elusive. By deleting Gpr177, we show that the Wnt-mediated alteration of MaSC properties and mammary cell type-specification are alleviated, leading to prevention of malignant lesions in MMTV-Wnt1 mice. Our findings provide an important link of stem cell alteration to the initiation of malignancy. They also provide a proof-of-principle for targeting of Gpr177 in diseases caused by aberrant Wnt stimulation.

Our results demonstrate that removal of a Wnt transcriptional target, Gpr177, prevents Wnt-induced mammary hyperplasia, providing a potential option for cancer prevention. Whether Gpr177 inhibition has therapeutic effects on cancer remains to be tested. Although Wnt overexpression may not be responsible for the hyperactive Wnt signaling in human breast cancers, targeting this pathway upstream of mutated APC or β-catenin has also been used since recent evidence suggest that such compounds might be beneficial [16]. In addition, targeting Gpr177 could be applicable to other human diseases associated with Wnt stimulation. Newly created inhibitors of Wnt/β-catenin signaling, including monoclonal antibodies and small interfering RNAs against Wnt proteins, have recently entered preclinical trials [54]. The targeting of Gpr177 to modulate Wnt production and signaling can also be developed as a new disease treatment.

## Supporting Information

Figure S1 **MMTV-Cre transgene induces site-specific recombination in mammary development.** β-gal staining in whole mounts (A–F) and sections (G–I) demonstrates the efficacy of Cre-mediated recombination mediated by MMTV-Cre at P0 (A), P7 (B, G), P14 (C), v3W (D), v1M (E, H) and v2M (F, I). Double labeling with β-gal staining in blue and immunostaining of the cellular marker in brown indicates that the Cre activity is detected in mammary cells positive for K18 (J, M), K14 (K, N) and SMA (L, O) in the duct (J–L) and TEB (M–O). Scale bars, 500 µm (A–F); 100 µm (G); 50 µm (H–O).
(DOC)

Figure S2 **Mammary morphogenesis is impaired by Gpr177 deficiency.** Whole staining shows defects in mammary development associated with inactivation of Gpr177 by MMTV-Cre (Gpr177^MMTV^) at P0 (A, B), P14 (C, D), and P21 (E, F). Broken lines highlight the mammary gland at P0. N, nipple. Scale bars, 500 µm (A–F).
(DOC)

Figure S3 **The control experiment shows specificity of the immunostaining analyses.** Sections of the v1M (A–C) and v2M (D–F) mammary glands were analyzed by immunostaining with only the goat (G2°Ab), rabbit (R2°Ab) or mouse (M2°Ab) secondary antibodies. No signals (brown) were detected in the epithelial cells except for non specific reactivity in the fatty area. Scale bar, 50 µm (A–F).
(DOC)
