# Supplementary material for: Gpr177 Deficiency Impairs Mammary Development and Prohibits Wnt-Induced Tumorigenesis
Source: PLoS One. 2013 Feb 15;8(2):e56644. doi: 10.1371/journal.pone.0056644 (PMC3574013; doi:10.1371/journal.pone.0056644)

**Supporting Information**

Figure S1. MMTV-Cre transgene induces site-specific recombination in mammary development. β-gal staining in whole mounts (A-F) and sections (G-I) demonstrates the efficacy of Cre-mediated recombination mediated by MMTV-Cre at P0 (A), P7 (B, G), P14 (C), v3W (D), v1M (E, H) and v2M (F, I). Double labeling with β-gal staining in blue and immunostaining of the cellular marker in brown indicates that the Cre activity is detected in mammary cells positive for K18 (J, M), K14 (K, N) and SMA (L, O) in the duct (J-L) and TEB (M-O). Scale bars, 500 μm (A-F); 100 μm (G); 50 μm (H-O).


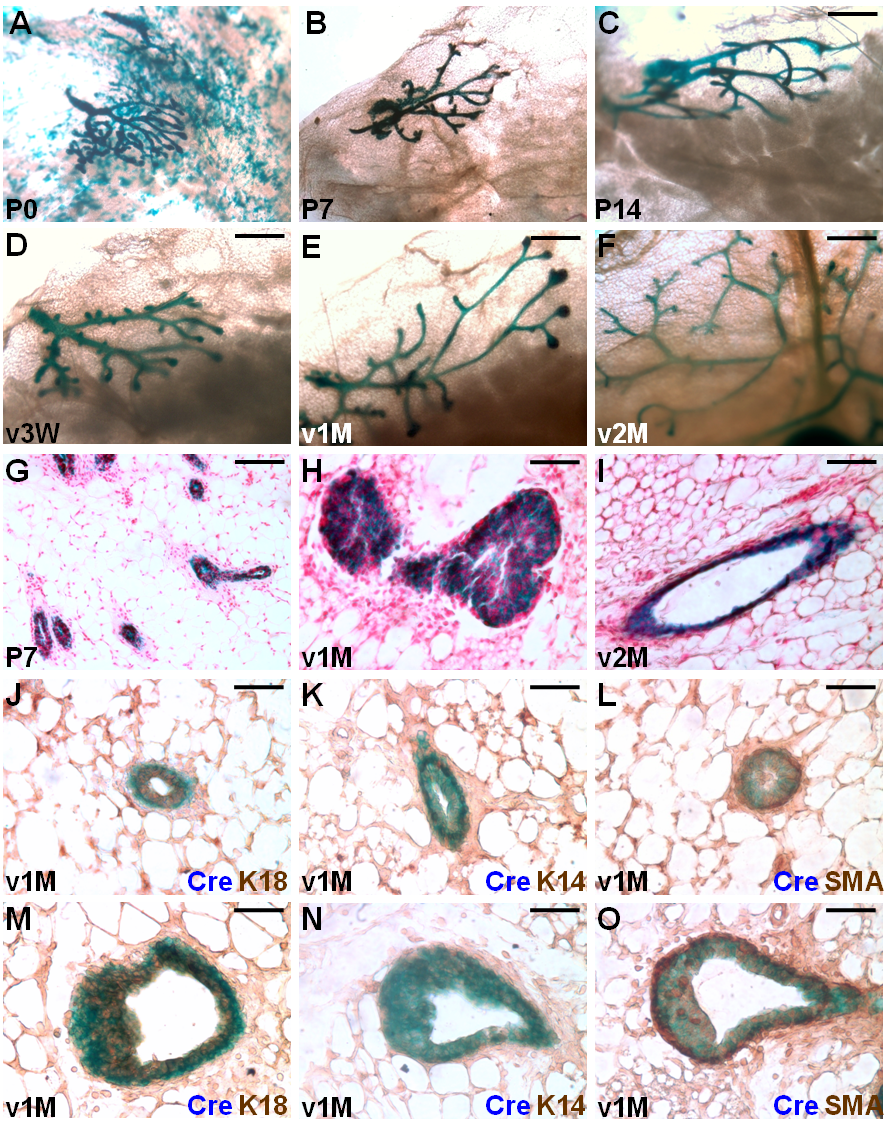

Supplement: Figure S1 — MMTV-Cre transgene induces site-specific recombination in mammary development. β-gal staining in whole mounts (A–F) and sections (G–I) demonstrates the efficacy of Cre-mediated recombination mediated by MMTV-Cre at P0 (A), P7 (B, G), P14 (C), v3W (D), v1M (E, H) and v2M (F, I). Double labeling with β-gal staining in blue and immunostaining of the cellular marker in brown indicates that the Cre activity is detected in mammary cells positive for K18 (J, M), K14 (K, N) and SMA (L, O) in the duct (J–L) and TEB (M–O). Scale bars, 500 µm (A–F); 100 µm (G); 50 µm (H–O). (DOC) [file pone.0056644.s001.doc]
