# Supplementary material for: Gpr177 Deficiency Impairs Mammary Development and Prohibits Wnt-Induced Tumorigenesis
Source: PLoS One. 2013 Feb 15;8(2):e56644. doi: 10.1371/journal.pone.0056644 (PMC3574013; doi:10.1371/journal.pone.0056644)

**Supporting Information**

Figure S2. Mammary morphogenesis is impaired by Gpr177 deficiency. Whole staining shows defects in mammary development associated with inactivation of Gpr177 by MMTV-Cre (Gpr177^MMTV^) at P0 (A, B), P14 (C, D), and P21 (E, F). Broken lines highlight the mammary gland at P0. N, nipple. Scale bars, 500 μm (A-F).


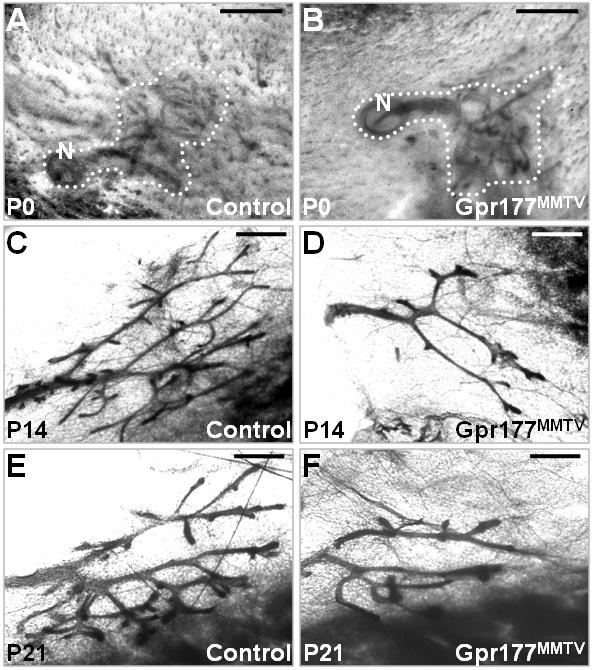

Supplement: Figure S2 — Mammary morphogenesis is impaired by Gpr177 deficiency. Whole staining shows defects in mammary development associated with inactivation of Gpr177 by MMTV-Cre (Gpr177MMTV) at P0 (A, B), P14 (C, D), and P21 (E, F). Broken lines highlight the mammary gland at P0. N, nipple. Scale bars, 500 µm (A–F). (DOC) [file pone.0056644.s002.doc]
