# Supplementary material for: Gpr177 Deficiency Impairs Mammary Development and Prohibits Wnt-Induced Tumorigenesis
Source: PLoS One. 2013 Feb 15;8(2):e56644. doi: 10.1371/journal.pone.0056644 (PMC3574013; doi:10.1371/journal.pone.0056644)

**Supporting Information**

Figure S3. The control experiment shows specificity of the immunostaining analyses. Sections of the v1M (A-C) and v2M (D-F) mammary glands were analyzed by immunostaining with only the goat (G2^o^Ab), rabbit (R2^o^Ab) or mouse (M2^o^Ab) secondary antibodies. No signals (brown) were detected in the epithelial cells except for non specific reactivity in the fatty area. Scale bar, 50 μm (A-F).


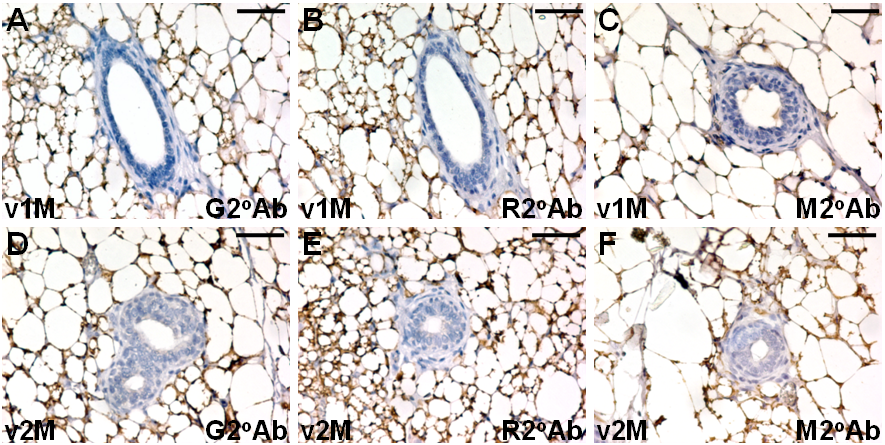

Supplement: Figure S3 — The control experiment shows specificity of the immunostaining analyses. Sections of the v1M (A–C) and v2M (D–F) mammary glands were analyzed by immunostaining with only the goat (G2°Ab), rabbit (R2°Ab) or mouse (M2°Ab) secondary antibodies. No signals (brown) were detected in the epithelial cells except for non specific reactivity in the fatty area. Scale bar, 50 µm (A–F). (DOC) [file pone.0056644.s003.doc]
